# PRISM 2.0: Technical Challenges

**DOI:** 10.1093/geroni/igab046.1193

**Published:** 2021-12-17

**Authors:** Neil Charness, Walter Boot, Sara Czaja, Wendy Rogers, Joseph Sharit

**Affiliations:** 1 Florida State University, Tallahassee, Florida, United States; 2 Weill Cornell Medicine/Center on Aging and Behavioral Research, New York, New York, United States; 3 University of Illinois Urbana-Champaign, Champaign, Illinois, United States; 4 Department of Industrial Engineering, University of Miami, Coral Gables, Florida, United States

## Abstract

PRISM 2.0 was designed to run on Android tablets and made use of both customized apps that relied on Google’s browser and e-mail functionality as well as commercial apps, such as Microsoft’s Skype for videoconferencing. We also made use of functionality provided by our partner AT&T, such as their sim cards to provide cell-based internet connectivity to participants who did not have access to Wi-Fi internet services to their home (cable, DSL), as well as tablet management software to deploy updates. The Miami site provided central management and tablet deployment and redeployment services and support as well as coordinating locally provided tech support at the three sites. We discuss some of the technical challenges associated with these arrangements. We focus on how changes to the operating system broke some of our apps necessitating substitution of other apps and provision of new training, and how Covid-19 affected technical support.

